# Physiological Action of Chloroform

**Published:** 1891-01

**Authors:** 


### PHYSIOLOGICAL ACTION OF CHLOROFORM.

TRANSLATED FROM "LE PROGRESS MEDICAL," OF PARIS, BY DR. VAN GOIDTSNOVEN, OF ATLANTA, GEORGIA.

The physiological action of chloroform is now one of the main topics of discussion in many societies of learning. This investigation has already occupied several meetings of the Academy of Medicine of Paris.

In England two commissions have published reports on this subject: the commission of Glasgow and the Hyderabad Chloroform Commission. The conclusions of the latter have been published in the London Lancet, June 21st, 1890, p. 1369.

As is well known, this commission is the second of that name and had, as the preceding one, the study of the accidents of chloroform as its main object; and was started under the patronage and liberality of the Nizam of Hyderabad. Its special mission was to study those accidents on purely physiological grounds, and to avail itself and take control of all the clinical observations of its predecessor.

Experiments were wrought on 430 animals with chloroform alone, and on 168 with both chloroform and ether. The respiratory movements of the chest, the actions of the heart and the pulse were registered throughout the whole course of investigations, Ludwig's manometer and Fick's kymograph being often resorted to.

The report shows that chloroform when administered without any interruption, produces a gradual fall of blood pressure and does not interfere with the free and easy respiration of the animal. If this fall of blood pressure be prolonged, the animal becomes insensible; then respiration undergoes a gradual diminution, and later on the heart's action ceases.

If chloroform be administered in heavy doses, death ensues more promptly, yet it invariably comes on gradually in relatively the same conditions as above specified. However concentrated the mixture of chloroform and air may be, it never causes sudden death through heart failure.

Chloroform does not increase tendencies to shock or syncope during operations. The members of the commission performed every possible operation on animals without observing any perturbation whether in the heart, the pulse or blood pressure. Struggles and movements made by the animal during inhalation, in a word anything of a nature to arrest respiration will produce irregularities of the circulation and of the heart's action. The act of drawing the tongue forward, which is done frequently when the patient is thought to be in danger, is, according to the commission's report, one of the main causes of shock, through possible irritation of the pneumogastric.

The electric stimulation of the vagus,far from being followed by a diminution of blood pressure, as has heretofore been thought, seems to have a beneficial result. Throughout the course of their experiments the commission have observed its good results whenever there was arrest of respiration at the beginning of the inhalations, or in asphyxia, or even when the respiratory centre was paralyzed in the last stage of chloroform administration.

The commission arrived progressively to this conclusion:

That chloroform can be administered until complete anæsthesia be effected, with a gradual fall of blood pressure, provided there be no irregularity in the heart's action. To secure a natural and regular respiration without resistance, suppression, asphyxia or any trouble in the respiratory act, should be the paramount endeavor, and the main object in view.

Resisting efforts render inhalations dangerous because the patient undergoes a partial asphyxia, and all his motions accelerate circulation and inhalation, thus increasing both the inhalation and absorption of chloroform. The nerve centers become then more rapidly affected. The momentary arrest of respiration causes asphyxia; and the latter, whether due to this or other causes, excites in its turn the respiratory centre. Respiration becomes deeper and more frequent and the patient absorbs more chloroform. The main defect attending inhalations is that they contain too much chloroform, the latter being poured too rapidly, and the patient absorbs too great a quantity of the narcotic; hence respiration and inhalation stop rapidly.

The best inhaling instrument is a cone in which the chloroform should be poured in small doses. This instrument should be first applied a small distance from the mouth and nose of the patient. If respiration continue without any difficulty, it should gradually be placed nearer until finally adjusted.

Thus it will be seen from the facts and data published by the Hyderabad commission, that respiration always stops before circulation, the heart being the last organ whose movements come to an end. One should not, therefore, put too much reliance in the pulse, since on the one hand the blood pressure begins to diminish long before the general anæsthesia is effected, and on the other the action of the heart ceases only after the circulation is arrested.

---

### PERITONITIS.

---

Dr. Emory Lanphear concisely sums up the desiderata in the treatment of peritonitis as follows :

The saline cathartic treatment should be adopted early in simple acute peritonitis.

Small doses of calomel may be given to mild purgation in cases seen after the disease is fully developed.

Cases which fail to be relieved by cathartic measures should receive early operative interference.

Whenever peritonitis has gone on to that stage where the formation of pus is known or even suspected to have taken place, abdominal section and drainage are imperatively indicated.

When the existence of tubercular peritonitis is diagnosticated, or strongly suspected, operation (explanatory incision) is justifiable.

Opium is only indicated in the second stage of peritonitis, and then not because it "forms a splint," but because it relieves pain, sustains the heart,, and prevents shock—thus combating the tendency of death.—*Med. Age.*

---

COPIES of an excellent lecture on sexual perversion, by G. Frank Lydston, M. D., of Opera House Block, Chicago, Ill., can be had by addressing the author.

